# P-1395. Safety and Effectiveness of LC16m8 for Pre-Exposure Prophylaxis Against mpox in High-Risk Population: An Open-Label Randomized Trial

**DOI:** 10.1093/ofid/ofae631.1571

**Published:** 2025-01-29

**Authors:** Nobumasa Okumura, Eriko Morino, Hidetoshi Nomoto, Mashiho Yanagi, Kozue Takahashi, Haruka Iwasaki, Yukari Uemura, Yosuke Shimizu, Daisuke Mizushima, Kazuaki Fukushima, Ei Kinai, Daisuke Shiojiri, Ichiro Itoda, Yasuhiko Onoue, Yoshitomo Kobori, Fukumi Nakamura, Daisuke Tokita, Wataru Sugiura, Norio Ohmagari, Mugen Ujiie

**Affiliations:** National Center for Global Health and Medicine, Shinjuku-ku, Tokyo, Japan; National Center for Global Health and Medicine, Shinjuku-ku, Tokyo, Japan; Disease Control and Prevention Center, National Center for Global Health and Medicine, Tokyo, Japan, Shinjuku-ku, Tokyo, Japan; National Center for Global Health and Medicine, Shinjuku-ku, Tokyo, Japan; National Center for Global Health and Medicine, Shinjuku-ku, Tokyo, Japan; National Center for Global Health and Medicine, Shinjuku-ku, Tokyo, Japan; National Center for Global Health and Medicine, Shinjuku-ku, Tokyo, Japan; National Center for Global Health and Medicine, Shinjuku-ku, Tokyo, Japan; National Center for Global Health and Medicine, Shinjuku-ku, Tokyo, Japan; Tokyo Metropolitan Cancer and Infectious Diseases Center Komagome Hospital, Arakawa-ku, Tokyo, Japan; Tokyo Medical University Hospital, Tokyo, Tokyo, Japan; Personal Health Clinic, Tokyo, Tokyo, Japan; Shirakaba Clinic, Tokyo, Tokyo, Japan; Private Care Clinic Tokyo, Shinjuku Site, Tokyo, Tokyo, Japan; Private Care Clinic Tokyo, Tokyo Site, Tokyo, Tokyo, Japan; Tokyo Metropolitan Bokutoh Hospital, Tokyo, Tokyo, Japan; National Center for Global Health and Medicine, Shinjuku-ku, Tokyo, Japan; National Center for Global Health and Medicine, Shinjuku-ku, Tokyo, Japan; National Centre for Global Health and Medicine, Shinjuku, Tokyo, Japan; 2. Center Hosp. of the Nat'l Ctr. for Global Health & Medicine, Tokyo, Tokyo, Japan

## Abstract

**Background:**

Since May 2022, the incidence of mpox cases has surged outside endemic regions. Although vaccination remains pivotal in mpox prevention, data on LC16m8, a third-generation smallpox vaccine, are scant. We provided LC16m8 pre-exposure prophylaxis opportunities to high-risk individuals, including those with HIV, and conducted a randomized controlled trial to assess LC16m8’s effectiveness in mpox prevention and safety.

**Figure:**
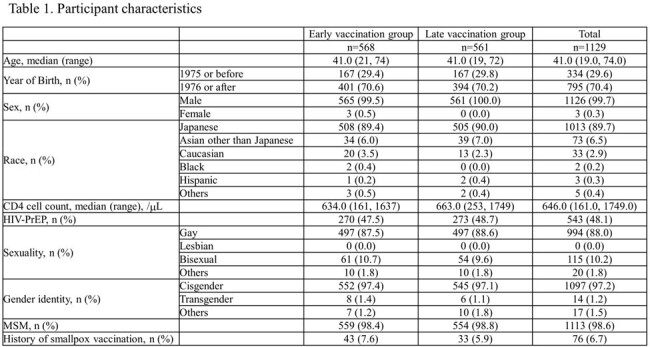

**Methods:**

Our multicenter randomized open-label trial enrolled men and women aged ≥18 years at high mpox risk who provided written consent. Participants were randomly assigned 1:1 to early or late vaccination groups, receiving vaccinations approximately 70 days apart. Primary endpoint: vaccine effectiveness (VE) against mpox development between early and late vaccinations. VE against severe mpox, symptoms, “take” incidence, and adverse events were secondary endpoints.

**Figure:**
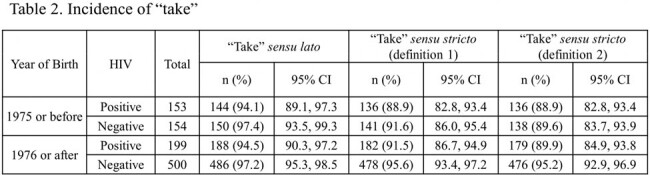

**Results:**

A total of 1,135 participants were recruited; 570 and 565 were assigned to early and late vaccination groups, respectively; 530 and 476 were vaccinated. Median age: 41 years; 99.7% were male; 89.7% Japanese; and 34.4% HIV-infected. No mpox cases occurred during the observation period, precluding VE calculation. “Take” rates: 90.3% (HIV-infected), 94.6% (uninfected). Adverse events related to the study were observed in 96.6% and 98.0% of the HIV-infected and uninfected participants, respectively. No fatal adverse events were observed; serious adverse events (SAE): 0.6% (HIV-infected), 0.5% (uninfected). One HIV-uninfected participant reported pulmonary embolism and deep vein thrombosis as a causally undeniable SAE. Local skin reactions: 96.6% (HIV-infected), 97.9% (uninfected); systemic reactions: 63.6% (HIV-infected), 64.2% (uninfected).

**Figure:**
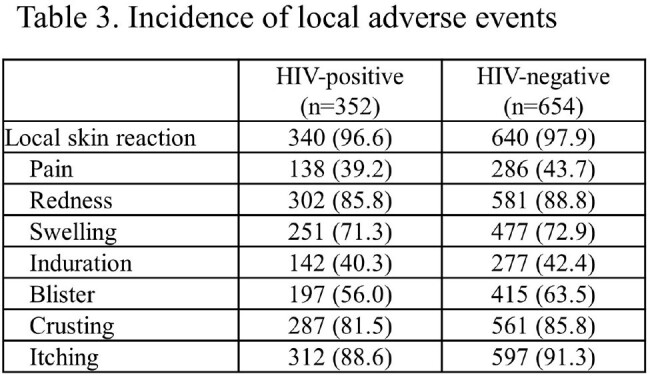

**Conclusion:**

LC16m8’s effectiveness in mpox prevention remains inconclusive. Yet, its use in well-controlled HIV-infected and -uninfected individuals showed no significant safety concerns, suggesting potential for targeted vaccination strategies in at-risk groups.

**Figure:**
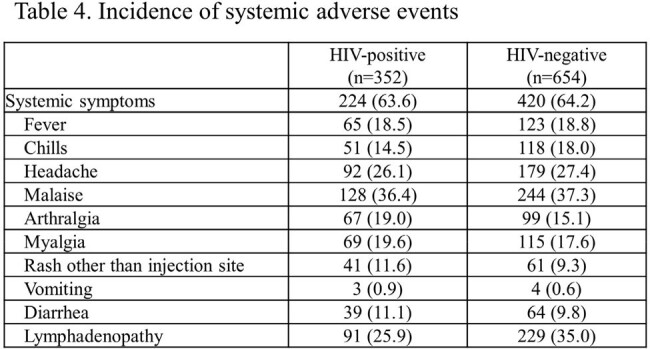

**Disclosures:**

**All Authors**: No reported disclosures

